# *HT-B* and *S-RNase* CRISPR-Cas9 double knockouts show enhanced self-fertility in diploid *Solanum tuberosum*

**DOI:** 10.3389/fpls.2023.1151347

**Published:** 2023-05-31

**Authors:** Sarah Lee, Felix E. Enciso-Rodriguez, William Behling, Thilani Jayakody, Kaela Panicucci, Daniel Zarka, Satya Swathi Nadakuduti, C. Robin Buell, Norma C. Manrique-Carpintero, David S. Douches

**Affiliations:** ^1^ Department of Plant, Soil and Microbial Sciences, Michigan State University, East Lansing, MI, United States; ^2^ Horticultural Sciences Department, University of Florida, Gainesville, FL, United States; ^3^ Enviromental Horticulture Department, University of Florida, Gainesville, FL, United States; ^4^ Department of Crop and Soil Sciences, Center for Applied Genetic Technologies, Institute for Plant Breeding, Genetics and Genomics, University of Georgia, Athens, GA, United States; ^5^ Alliance of Bioversity International and The International Center for Tropical Agriculture (CIAT), Cali, Colombia

**Keywords:** Self-incompatibility, *S-RNase*, *HT-B*, *Solanum tuberosum*, CRISPR-Cas9, diploid potato breeding

## Abstract

The Gametophytic Self-Incompatibility (GSI) system in diploid potato (*Solanum tuberosum* L.) poses a substantial barrier in diploid potato breeding by hindering the generation of inbred lines. One solution is gene editing to generate self-compatible diploid potatoes which will allow for the generation of elite inbred lines with fixed favorable alleles and heterotic potential. The *S-RNase* and *HT* genes have been shown previously to contribute to GSI in the Solanaceae family and self-compatible *S. tuberosum* lines have been generated by knocking out *S-RNase* gene with CRISPR-Cas9 gene editing. This study employed CRISPR-Cas9 to knockout *HT-B* either individually or in concert with *S-RNase* in the diploid self-incompatible *S. tuberosum* clone DRH-195. Using mature seed formation from self-pollinated fruit as the defining characteristic of self-compatibility, *HT-B-*only knockouts produced little or no seed. In contrast, double knockout lines of *HT-B* and *S-RNase* displayed levels of seed production that were up to three times higher than observed in the *S-RNase*-only knockout, indicating a synergistic effect between *HT-B* and *S-RNase* in self-compatibility in diploid potato. This contrasts with compatible cross-pollinations, where *S-RNase* and *HT-B* did not have a significant effect on seed set. Contradictory to the traditional GSI model, self-incompatible lines displayed pollen tube growth reaching the ovary, yet ovules failed to develop into seeds indicating a potential late-acting self-incompatibility in DRH-195. Germplasm generated from this study will serve as a valuable resource for diploid potato breeding.

## 1 Introduction

Potato (*Solanum tuberosum* L.) represents one of the most economically important species within the Solanaceae family along with tomato, eggplant, pepper, tobacco and petunia (Olmstead et al., 2008; Wu and Tanksley, 2010). Potato is the world’s third most important food crop (Devaux et al., 2014) contributing to global food security with an annual production of over 376 million metric tons (Faostat, 2023). Commercial potato varieties are heterozygous, autotetraploid, and experience acute inbreeding depression when self-pollinated over multiple generations which complicates traditional breeding schemes (Jansky et al., 2016; Zhang et al., 2019). Shifting potato production from tetraploid clonal propagation to diploid self-compatible reproduction has several advantages including a shorter breeding cycle, introgression of advantageous alleles from wild diploid potato relatives, and the generation of inbred lines (Lightbourn and Veilleux, 2007; Jansky et al., 2016; Bradshaw, 2022). The generation of inbred lines is of particular interest as this is the most direct approach for yield improvement *via* heterosis, increasing genetic gain, and fixing favorable allelic combinations as seen in grain crops such as maize (*Zea mays* L.) (Hosaka and Hanneman, 1998; Jansky et al., 2016). However, the self-incompatible nature of most diploid potatoes is a significant barrier to the generation of inbred diploid potato lines.

Self-pollen rejection in potatoes is controlled by the gametophytic self-incompatibility (GSI) system, which is present mainly in the Solanaceae and Rosaceae families (Kondo et al., 2002; Takayama and Isogai, 2005; Sassa et al., 2007; Boivin et al., 2014). The GSI model asserts that factors from both the female determinant (S-RNase in the style) and male determinant (S-locus F-Box proteins [SLFs] from the pollen) contribute to self-incompatibility (SI) (McClure et al., 1989; McClure et al., 1990; Sijacic et al., 2004; McClure, 2006; Kubo et al., 2010). In cross pollinated plants, the SLF proteins prevent S-RNase-mediated pollen RNA degradation *via* a proteosome system, acting as a detoxification mechanism (Takayama and Isogai, 2005; Kubo et al., 2010; McClure et al., 2011). Detoxification will not occur if the S-haplotype between the pollen and the style match, as a consequence, self-pollinated plants with matching haplotypes of SLF and S-RNase are not fertilized (McClure et al., 1989; McClure et al., 1990; Takayama and Isogai, 2005; McClure et al., 2011). Currently, diploid breeding programs use native genetic sources to remove SI barriers. The dominant *Self incompatibility inhibitor* locus (*Sli*) from self-compatible *S. chacoense* lines has been well documented (Hosaka and Hanneman, 1998; Jansky et al., 2014; Jansky et al., 2016; Clot et al., 2020). This locus contains a gene encoding a non-*S*-locus F-Box protein that acts by inhibiting self-*S-RNase* allelic variants (Eggers et al., 2021; Ma et al., 2021). M6, an inbred *S. chacoense* line, has been a common source of the *Sli* locus in diploid potato breeding (Jansky et al., 2014; Clot et al., 2020). In some instances, the introgression of *Sli* into cultivated diploid materials has allowed breeders to overcome SI and shift potato breeding towards the generation of inbred lines (Alsahlany et al., 2021). However, introgression of *Sli* from M6 into other germplasm is time-consuming and could lead to linkage drag and fixation of undesirable traits such as high tuber glycoalkaloid content and other inferior tuber traits (Jansky et al., 2014). Furthermore, other undefined factors may be associated with self-compatibility (SC) mechanism or interact with the *Sli*-based SC during inbreeding, adding confounding effects to obtain self-compatible plants through traditional breeding (Kaiser et al., 2021)

To avoid the introgression of undesirable genes through conventional breeding, targeted mutagenesis using Clustered Regularly Interspaced Short Palindromic Repeats (CRISPR)-associated protein 9 (Cas9) gene editing may be used for gene targeting to alter specific traits (Nadakuduti et al., 2018; Pixley et al., 2022). CRISPR-Cas9 has already proven to be a viable option for generating self-compatible plants, as demonstrated with the knockout (KO) of *S-RNase* (Ye et al., 2018; Enciso-Rodriguez et al., 2019). In the *S-RNase* KO study reported by Enciso-Rodriguez et al. (2019), plasticity in both gene edited and non-edited lines was observed in SI and fertility (fruit and seed count).

Though *S-RNase* is a major modulator of SI in potato, there are other contributing factors and/or modifier genes such as HT proteins previously reported in the Solanaceae family (McClure et al., 1999; O’Brien et al., 2002; Goldraij et al., 2006; Tovar-Méndez et al., 2017; Broz and Bedinger, 2021). In a hypothesized sequestering model, S-RNase and HT-B proteins undergo endocytosis from the extracellular matrix (ECM), encapsulating S-RNase in a vacuolar compartment (Goldraij et al., 2006; Tovar-Méndez et al., 2017). Immunolocalization experiments suggest that HT-B is present on the vacuolar membrane containing S-RNase. HT-B is degraded in self-compatible pollen tubes sequestering S-RNase, yet remains functional in incompatible pollen tubes (Goldraij et al., 2006; McClure et al., 2011). Degradation of HT-B is associated with stability of the vacuolar membrane, and only occurs if the *S*-haplotypes between the style and the pollen are dissimilar (Goldraij et al., 2006; McClure et al., 2011). Alternatively, if the *S*-haplotypes are identical, HT-B will remain intact, yet the vacuolar membrane is degraded leading to release of the S-RNase and inhibition of fertilization (Goldraij et al., 2006; McClure et al., 2011). This sequestering model is based on studies in tobacco (*Nicotiana alata)* and a wild potato relative (*S. chacoense*) in which both species displayed SC when the *HT-B* gene was suppressed *via* RNA interference (RNAi) (McClure et al., 1999; O’Brien et al., 2002). Another *HT* gene, *HT-A*, was suppressed in *S. chacoense* but did not result in SC (O’Brien et al., 2002).

These genetic factors, in concert with environmental parameters and ontogeny, influence the SI response, which can show plasticity in fertility metrics such as fruit set (Webb and Williams, 1988; Stone et al., 2006; Mena-Ali and Stephenson, 2007; Enciso-Rodriguez et al., 2019). Generally, lower fruit set is observed at higher temperatures and the SI response breaks down as the plant matures (Webb and Williams, 1988; Travers et al., 2004; Stone et al., 2006; Mena-Ali and Stephenson, 2007; Enciso-Rodriguez et al., 2019). This study seeks to understand the role of *HT-B* in *S. tuberosum* by targeting *HT-B* for KO either individually or in addition to *S-RNase* using CRISPR-Cas9-mediated gene editing, while also considering environmental factors and plant age. In addition to enhancing our understanding of the SI response in *S. tuberosum*, self-compatible lines resulting from gene edits may be used as a resource for SC in reinventing potato as a diploid crop.

## 2 Material and methods

### 2.1 Plant material

The SI diploid potato line DRH-195 (2*n*=2*x*=24), generated from a cross between *S. tuberosum* Gp. Phureja DM 1-3 516 R44 (DM) and *S. tuberosum* Gp Tuberosum RH 89-039-16 (RH), was selected for this study. DRH-195, based on single nucleotide polymorphism analyses, has been shown to be *Sli* negative (Kaiser et al., 2021). DRH-195 derived *S-RNase* KO line (DRH-195.158) from a previous study (Enciso-Rodriguez et al., 2019) was used for comparison in this study. The SI line MSDD829-09 was used to determine seed set in compatible cross pollinations. The DRH-195 line (hereafter referred to as wild type - WT), KO and MSDD829-09 lines were propagated *in vitro* on Murashige and Skoog (MS) medium (MS basal salts plus vitamins, 3% sucrose, 0.7% plant agar, pH 5.8) (Murashige and Skoog, 1962). *In vitro* plants were maintained in growth chambers with 16-h-light/8-h-dark photoperiod at 22°C and average light intensity of 200 μmoles m^-2^s^-1^.

### 2.2 Primer design, amplification, and sequencing of *HT-B*

DNA was isolated from young leaves of DRH-195 using the DNeasy Plant Mini Kit (Qiagen, Hilden, Germany) and used for PCR with a Q5^®^ High-Fidelity DNA Polymerase (New England Biolabs, Ipswich, MA, United States). *HT-B* primers were designed from conserved regions of reported *HT-B* gene/cDNA sequences retrieved from the National Center for Biotechnology Information nucleotide database (**Table S1**), and amplified using the following thermocycler conditions: one cycle of initial denaturation for 4 min at 94°C, followed by 34 cycles for 15 s at 30°C, 45 s at 56°C and 45 s at 72°C and a final extension of 5 min at 72°C. Amplicons were gel-purified using the QIAquick PCR Purification Kit (Qiagen, Hilden, Germany). Purified amplicons were A tailed, cloned into the pGEM T-Easy cloning vector (Promega, Madison, WI, United States) and transformed into DH5α competent cells (Thermo Fisher, Carlsbad, CA, United States). Finally, 40 colonies were Sanger sequenced and aligned using Clustal Omega (Sievers and Higgins 2014).

### 2.3 Single guide RNA (sgRNA) selection, assembly, and validation

Single guide RNAs were designed from the *HT-B* ORF, using the CRISPR RGEN tools (Park et al., 2015) and assembled using the Golden Gate cloning method in a T-DNA binary vector (pHSE401) carrying the Cas9, U6 promoter and scaffold guide RNA as described by Xing et al. (2014). Two separate constructs were generated to target different *HT-B* gene regions containing the sgRNA-HTB1 (pSPUD-121) and sgRNA-HTB2 (pSPUD-123), respectively. Additionally, a two guide RNA construct was assembled using the previously reported sgRNA-1 to the *S-RNase* gene (Enciso-Rodriguez et al., 2019) and the sgRNA-HTB1 (pSPUD-124). Finally, each assembled construct was transformed into *Agrobacterium tumefaciens* strain GV3101, carrying the nopaline type Ti plasmid pMP90, as described by Enciso-Rodriguez et al. (2019). On-target activity of the reagents was validated using DRH-195 protoplasts by tagging a double-stranded oligodeoxynucleotide (dsODN) into the CRISPR-Cas9 reagents induced double-strand breaks in the *HT-B* and *S-RNase* genes as described by Nadakuduti et al. (2019). PCRs amplifications were performed to detect the presence of dsODN at the target site on both genes.

### 2.4 *HT-B/S-RNase* knockout and screening of gene-edited events

DRH-195 leaf segments from 4-week-old tissue culture plants were used for *Agrobacterium*-mediated transformation as described by Enciso-Rodriguez et al. (2019). DNA from leaf tissue of plantlets from rooted transformation events (T_0_) was isolated using the DNeasy Plant Mini Kit (Qiagen, Hilden, Germany), and PCR-screens were performed using GoTaq Green Master Mix (Promega, Madison, Wisconsin) and the following thermocycler conditions: one cycle of initial denaturation for 5 min at 95°C, followed by 35 cycles for 15 s at 95°C, 45 s at 56°C and 45 s at 72°C and a final extension of 5 min at 72°C. Amplicons were viewed on a 2% agarose gel stained with SYBR-safe (Thermo Fisher Scientific, USA) and lines with noticeably large deletions were selected for this study. Lines with not visible deletions in agarose gels were subjected to a restriction enzyme (RE) digestion using *Hpy*CH4V (sgRNA-HTB1) and *Sml*I (sgRNA-HTB2) (New England Biolabs, USA). RE digest screening could not be performed on *S-RNase* target regions due to lack of cut sites near the protospacer adjacent motif (PAM) region. Candidate lines were identified and underwent additional PCR using Q5^®^ High-Fidelity DNA polymerase (New England Biolabs, USA) and subsequent Sanger Sequencing and analyzed as described previously. From the initial candidate lines, 14 WT, *S-RNase* KO, and selected T_0_ lines were transferred from subculture into 10 cm diameter pots, then were transferred 3 weeks later to 7.6L plastic pots with a peat and perlite growth medium mixture (Bacto professional plating mix, Houston, Texas). Plants were grown in a greenhouse with light intensity of 250 µmoles m-^2^s^-1^, 16/8-h light/dark photoperiod and a temperature of 25°C, through the months of late September – December in East Lansing, MI. Plants were fertilized biweekly with Peters Professional 20:20:20 fertilizer (The Scotts, Co., Marysville, OH, United States) and watered regularly.

### 2.5 Self-pollination, pollen viability, and ploidy determination

Flowers were self-pollinated using mature pollen from at least four flowers from the same plant. To prevent possible cross pollination, all flowers pollinated in this study were covered with a fine mesh bag before anthesis, contained in a separate section of the greenhouse, and self-pollinated within a day of anthesis. Pollen viability was determined *via* germination and acetocarmine staining as reported by Ordoñez (2014b). Briefly, pollen was collected in 1.5mL Eppendorf tube and mixed *via* gentle pipetting with 50uL of germination solution [22.5 mg Sucrose + 12.5 mg Boric Acid + 18.4 mg CaCl_2 +_ 29.5 mg Ca (NO_3_)_2 +_ 123 mg MgSO_4_/250 mL distilled water] and then placed on a glass slide. After 24 hours, 20uL of acetocarmine was added to the slide and pollen tube growth was verified under 10X magnification with a brightfield microscope (**Figure S1**). Tetraploid potatoes have an expected average of 12-14 chloroplasts per guard cell whereas diploids have an average of 6-8 chloroplasts per guard cell; to determine ploidy of all lines used in this study, chloroplasts were counted in 10 guard cells as described by Ordoñez (2014a), and lines with > 8 average chloroplasts were removed from the study (**Figure S2**).

### 2.6 Aniline blue pollen tube stain and visualization

Ten flowers for each WT, *S-RNase* KO, T_0_ KO lines were self-pollinated for stylar squash analysis. Within 48 hrs post-pollination a minimum of six carpels were immediately submerged in 750 uL of a 3:1 100% ethanol:glacial acetic acid fixation solution and incubated for at least 24 hrs in the dark at room temperature. The fixation solution was removed, and carpels were then submerged in 750 uL of 6 M NaOH solution and left overnight at room temperature. The softening solution was removed and washed three times with distilled H_2_O. The H_2_O was removed and then 1 mL of 0.1% aniline staining solution [100mg aniline blue + 760 mg K_3_PO_4 +_ 100 mL distilled H_2_O] was added. Tubes were wrapped in tin foil and incubated while shaking gently for 1 hour. Carpels were gently squashed on a glass slide with a cover slip and viewed with Nikon Imaging Software under 4X magnification using a DAPI filter at 358 nm wavelength.

### 2.7 Self-compatibility assessment and statistical analysis

Fruit set, fruit weight, and seed count served as quantitative measures for comparing self-fertility in the WT, *S-RNase* KO, and 14 T_0_ KO lines. The 14 lines were clonally propagated four times in tissue culture to give a total of 56 plants which were organized into a Randomized Complete Block Design. From each of the 56 plants, 15 flowers were self-pollinated. To account for possible changes in self-compatibility due to plant age, pollinations were repeated at one-month-old, and two-months-old, respectively. At each time point, 840 flowers were pollinated giving 1,680 total flowers pollinated within this study. Fruit set, fruit weight, and seed count data were recorded for each plant at each stage. ANOVA (alpha = 0.05) and Tukey pairwise comparison (alpha = 0.05) tests were performed in R using the glmmTMB (Brooks et al., 2017), lme4 (Bates et al., 2015), emmeans (Length, 2019), and multcompView (Graves et al., 2015) packages. Within this study, self-compatible plants are defined by both fruit and seed set. Seed count was used to define self-fertility as low, moderate, and high by having 1-20, 21-50, or > 50 seeds per fruit on average, respectively.

## 3 Results

### 3.1 Characterization of the *HT-B* gene in potato

**Figure 1:**
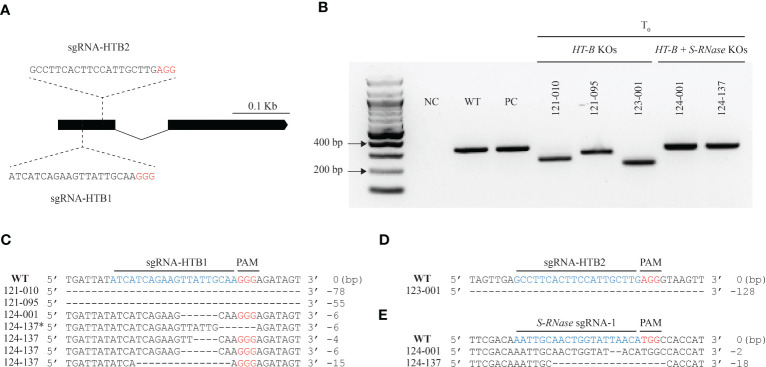
CRISPR-Cas9 gene-based mutagenesis of the *HT-B* gene. **(A)** The gene architecture of *HT-B* and location of two sgRNAs belonging to separate constructs designed to target conserved regions on exon 1. **(B)** *HT-B* edits compared to the WT and *S-RNase* KO which have no *HT-B* edits. Deletions of > 30bp were observed in 121_010, 121_095, and 123_001, one small < 10bp deletion (124_001), and one chimeric line (124_137) with multiple chimeric alleles (124_137*). Small deletions in the *S-RNase* KO, 124_001, and 124_137 lines are not noticeably different from the WT or *S-RNase* KO which have no *HT-B* gene edits. **(C)** DNA sequences of representative *HT-B* deletion products from the 121 lines resulting from sgRNA-HTB1 targeting. **(D)** A single line (123_001) with a large deletion in the *HT-B* gene resulting from sgRNA-HTB2 targeting. **(E)** Representative *S-RNase* edits. All lines are homozygous. **(C, D, E)** Deletions are represented by dashes. PC, positive control.

Primers were designed from conserved *HT-B* regions of multiple *Solanum* spp. and used to perform rapid amplification of cDNA ends (RACE) to amplify the *HT-B* open reading frame (ORF) from DRH-195. The DRH-195 *HT-B* gene is 401 bp (**Figure 1A**) and contains two exons and one intron with a predicted protein of 101 amino acids with a C-terminal Asn/Asp rich motif. The predicted HT-B potato protein is highly similar (79%, sequence ID: BAC00942.1) to a reported HT protein from *S. peruvianum*, a wild tomato relative (**Figure S3**). However, the potato HT predicted peptide lacks the first three amino acids (AFN) at the N-terminus (**Figure S3**). A similar trend is observed in HT sequences reported for most wild relatives of potato (*S. chacoense, S. bulbocastanum* and *S. pinnatisectum*) where only partial cDNA sequences are available, lacking up to 17 amino acids from the start codon relative to HT-B peptide sequences from the tomato clade. BLASTn analysis using the potato reference genome sequence (DM v6.1) (Pham et al., 2020) revealed that *HT-B* is located on chromosome 12 (chr12: 6563673- 6564073), close to the predicted *HT-A* gene (chr12: 6566633- 6567482). DRH-195 is a F_1_ progeny from a cross between the sequenced DM 1-3 516 R44 (DM) and RH-89-039-16 (RH) clones.

### 3.2 Targeted mutagenesis of *HT-B* and *S-RNase* using CRISPR-Cas9

Three separate constructs, pSPUD-121, pSPUD-123, and pSPUD-124 were used in this study. *HT-B* KOs were produced with two separate constructs, each containing a unique gRNA (sgRNA-HTB1 in construct pSPUD-121 or sgRNA-HTB2 in pSPUD-123) which targeted 44 bp or 77 bp downstream of the start codon in exon 1 of the *HT-B* gene, respectively (**Figure 1A**). The sgRNA targeting *S-RNase* designated as sgRNA1 and is described in Enciso-Rodriguez et al. (2019) was assembled along with sgRNA-HTB1 in construct pSPUD-124. The functionality of each construct was evaluated by transfecting wild type DRH-195 protoplasts along with dsODNs. Successful gene-editing reagents will induce a double stranded break (DSB) in the target region facilitating the dsODN integration, detected by PCR (Nadakuduti et al., 2019). Each sgRNA combination was able to generate DSBs and dsODNs were incorporated at each *HT-B* and *S-RNase* targeted regions in either, 5**’** to 3**’** or 3**’** to 5**’** direction. No dsODNs were detected in the negative control in which no reagent plasmid was used in the transfection (**Figure S4**).

After confirming edits in protoplasts, over 1000 DRH-195 leaf explants were used for *Agrobacterium*-mediated transformation. A 67%, 55%, and 42% of shoots were recovered from explants on selective media for the pSPUD-121, pSPUD-123, and pSPUD-124 constructs, respectively (**Table S2**) which are similar to results observed in the study by Enciso-Rodriguez et al. (2019) using DRH-195. The regenerated lines were screened using a combination of PCR gel electrophoresis and restriction enzyme digest assays for *bona fide* gene edits. The wild type DRH-195 and self-compatible *S-RNase* KO (DRH-195.158) generated by Enciso-Rodriguez et al. (2019) were used to compare and identify mutation events (**Figure 1B**). A total of 23 candidate lines for the three constructs were advanced for further study after screening over 300 lines. From the 23 candidate lines, 12 were confirmed to have gene edits using Sanger sequencing and were included with a non-edited and *S-RNase* KO line for downstream analyses.

### 3.3 CRISPR-Cas9 editing events result in *HT-B* and *HT-B + S-RNase* Gene KOs

CRISPR-Cas9 editing produced *HT-B* and/or *S-RNase* KOs by disrupting the gene(s) targeted. Representative events from *HT-B* and *HT-B* + *S-RNase* KO lines are shown in **Figures 1B-E**, sequences from all 12 lines are shown in **Figure S5** and compared to positive control sequences. The seven lines with mutations at *HT-B* gene generated with the sgRNA-HTB1 construct were labeled with “121” as prefix, the unique lines with deletions from sgRNA-HTB2 as “123”, and the four lines with successful edits in both the *HT-B* and *S-RNase* genes we named with the initial digits “124”. All edits of *HT-B* and *S-RNase* in T_0_ lines occurred near the PAM sequence in exon 1 of each gene (**Figure S5**). Most edits for selected *HT-B* and *S-RNase* lines were homozygous, while multiple *HT-B* allelic chimeric (three differing edits) mutations were found in the 121_008, 121_020, and 124_137 lines (**Figure S5**; **Table S3**). Regarding edits in the *HT-B* gene, a 1 bp insertion was recovered in the 121_005 line with the other mutations ranging from a small 1 bp deletion (121_008_C4) to several > 50 bp deletions (121_010, 121_062, 121_095, and 123_001) (**Figure S5**). The largest deletion observed was 128 bp (**Figure 1D**). Most lines with small *HT-B* deletions produced a frameshift mutation resulting in a premature stop codon or disrupted the reading frame likely resulting in a dysfunctional protein. The only exceptions were for two *HT-B* alleles with deletions that did not exhibit a frame shift mutation in the chimeric lines 121_020_C3 and 124_137_C4 (**Figure S5**). All *S-RNase* edits produced frameshift mutations which altered the predicted amino acid sequence.

### 3.4 Ploidy determination and pollen viability

Endoreduplication can occur in regenerated potatoes (Karp et al., 1984; Owen et al., 1988). Furthermore, tetraploid events are self-compatible and would confound the SC assessment in this study (Nettancourt, 1997; McClure et al., 2011; Campos and Ortiz, 2020). Therefore, the number of chloroplasts in guard cells in the T_0_ lines were counted to determine ploidy, with 8 chloroplasts set as cut off to be called as diploid, above which is polyploid (Karp et al., 1984). Out of the 32 lines selected for initial screening, 25% exhibited more than 8 chloroplasts on average, due to a possible chromosome doubling and were excluded from this study. From the remaining 24 lines, 14 lines with an average of 6-8 chloroplasts per guard cell indicating diploidy were chosen for this study (**Figure S2**). To ensure any variation in fertility was not due to male sterility, pollen from each gene edited line were stained with acetocarmine; all lines produced viable pollen and exhibited pollen tube growth (**Figure S1**).

### 3.5 Stylar squash analysis shows complete pollen tube growth in all lines

**Figure 2:**
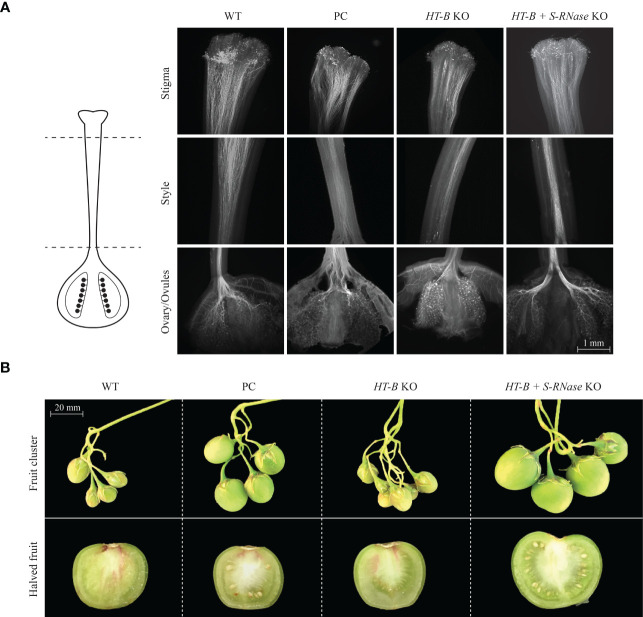
Qualitative assessment of pollen tube growth, fruit size, and seed count. **(A)** Pollen tube growth stained with aniline blue. Successful pollen tube growth from the stigma to the ovary in the WT, *S-RNase* KO, *HT-B* KO, *HT-B* + *S-RNase* KO lines. Samples above are representative of fourteen lines and 42 styles viewed. **(B)** Fruit size comparison is to scale; halved fruit are enlarged and not to scale. Fruit shown is representative of the most common phenotype observed for the WT, *S-RNase* KO, *HT-B* KO, *HT-B* + *S-RNase* KO lines. The *HT-B* and the *HT-B* + *S-RNase* samples are from the 121_020 and 124_137 lines, respectively. Parthenocarpic fruit shown for WT and *HT-B* KO lines. Seed was produced in the *S-RNase* KO and in the *HT-B* and *S-RNase* KO lines. Parthenocarpic fruit is noticeably smaller than seed producing fruit. PC = Positive control.

Three self-pollinated styles for each of the 14 selected lines (WT, *S-RNase* KO and 12 accessions generated in this study) were viewed under an epifluorescent microscope using aniline staining. As observed for the WT and *S-RNase* KO, all 14 lines showed pollen tube growth from the stigma through the style down to the ovaries (**Figure 2A**). The one exception was a single replication of 121_008 which lacked any pollen tube growth. Pollen tube growth from the stigma to the ovary and retention of SI is a form of Late-Acting Self-Incompatibility (LSI) and has previously been observed in other angiosperm species (Seavey and Bawa, 1986); the pollen-stylar interaction observed within this study may be due to LSI.

### 3.6 Double KO lines display self-compatibility and enhance self-fertility

**Figure 3:**
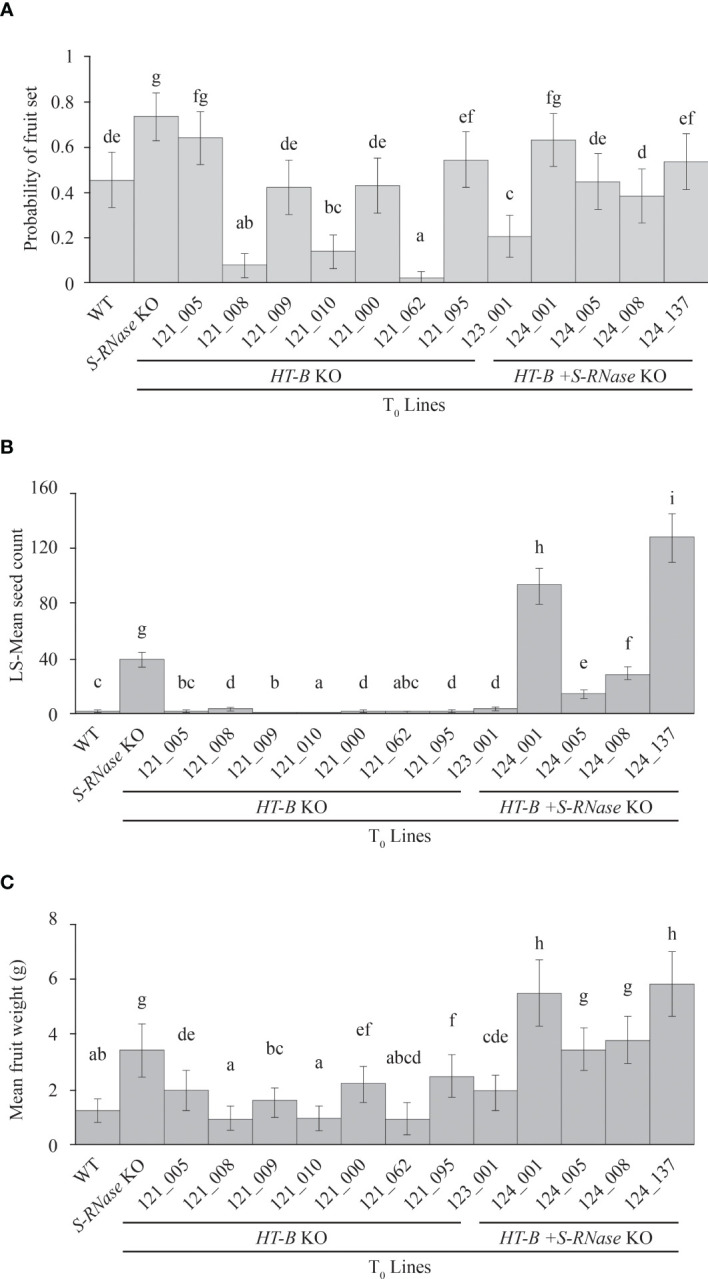
Quantitative analyses of self-fertility. **(A)** Significant differences in fruit set (p < 0.05, Tukey’s test) were observed between different lines, but there are no consistent trends in fruit set when comparing *HT-B*, *HT-B* + *S-RNase*, *S-RNase* KO, and the WT. **(B)** Fruit weight is significantly higher (p < 0.05, Tukey’s test) in self-compatible fruits with *HT-B + S-RNase* KO lines together with *S-RNase* KO compared to the WT or *HT-B* only knockouts. **(C)** Double knockout lines 124_001 and 124_137 have significantly higher seeds (p < 0.05, Tukey’s test), than the *S-RNase* KO. The WT and *HT-B* KOs had significant lower seed count (p < 0.05) with < 5 seeds per fruit on average. (a/b/c/d) Lines with the same letter are not significantly different from one another. PC, Positive control.

Quantitative analysis of self-fertility was performed using fruit set, fruit weight, and seed count analysis. Fruit set occurred in all lines including the self-incompatible WT, indicating that fruit set is not necessarily indicative of SC in this study (**Figure 2B**). For example, out of all 14 lines, 121_005 had the second highest frequency of setting fruit (64.0% +/- 6% SE) and yet only had a mean seed per fruit of 0.56 +/- 0.10 SE (**Figures 3A, B**). As seen in **Figure 3C**, *HT-B* + *S-RNase* KOs and the *S-RNase* KO had significantly greater fruit weight than the WT and *HT-B* KO lines (P < 0.05, Tukey’s test) that was visibly noticeable (**Figure 2B**). Interestingly, there was a significant increase in fruit weight between plants pollinated at one month and two and a half months (p < 0.05, ANOVA), but no significant relationship between plant age and seed count was observed (p > 0.05, ANOVA). The WT and *HT-B* KO lines exhibited less than three seeds per fruit, the *S-RNase* KO produced a moderate seed count (38.9 +/- 2.8 SE), and the *HT-B* + *S-RNase* KOs ranged from 14 – 128 seeds (**Figure 3C**). The *HT-B* + *S-RNase* KO line (124_005) with the lowest seed count (13.9 +/- 1.1 SE) also had severe floral mutations (**Figure S6**). The 124_008 line did not show enhanced fertility in comparison to the *S-RNase* KO line. The 124_001 and 124_137 lines (*HT-B* and *S-RNase* KOs) had up to three times greater seed count than the *S-RNase* KO. To further confirm the effect of the double KO in DRH-195, cross pollinations were made between four female parents the WT, a S-RNase KO (195.158), a HT-B KO (121_009), and a double KO (124_001); and a SI diploid breeding line (MSDD829-09) as the male parent. Average seed count per fruit for each of the four female lines was above 90 seeds. The three KO lines was not significantly different from each other 195.198 (104.9 +/- 2.9 SE), 121_009 (112.5 +/- 4.6 SE) and 124_001 (93.5 +/- 4 SE). Seed set in the WT (183 +/- 9.7 SE) was significantly higher than each of the KO lines, indicating a loss in female fertility in the transformed events. This highlights the impressive nature of the self-pollinations, where the increase in self-compatibility completely eclipsed any loss in female fertility.

## 4 Discussion

Converting potato breeding from a tetraploid clonally propagated platform to a self-compatible diploid breeding system has far reaching advantages from increased genetic gain potential as well as sexual reproduction that will lower the cost of storage by using true seed instead of vegetative tuber seeds (Howard, 1975; Jansky et al., 2016; Bradshaw, 2022). In this study, double KO lines with higher levels of self-fertility than previous *S-RNase* KO lines (Enciso-Rodriguez et al., 2019) were generated. This increase in self-fertility was also dramatic enough to extinguish any loss in female fertility in the KO lines that was observed in compatible cross pollinations Prior studies have described SC in diploid potato as a qualitative measurement defined in terms of presence, absence, or displaying pseudo SC (Peterson et al., 2016; Ye et al., 2018; Enciso-Rodriguez et al., 2019). While a qualitative viewpoint may be sufficient depending on the application, it may not accurately describe variation in SC and fertility often observed in *Solanum* species (Cipar et al., 1964). Rather than qualitatively describing SC/SI, this study quantitatively defines fertility by correlating the fruit weight, and seed count that may be anticipated from *S-RNase, HT-B*, and *S-RNase* + *HT-B* KO(s).

Using fruit set and weight as metrics of self-fertility, this study shows all self-pollinated lines produced fruit to varying degrees. DRH lines have shown plasticity in fruit set in previous studies (Peterson et al., 2016; Enciso-Rodriguez et al., 2019) and variation may occur due to environmental effects, such as temperature, photoperiod, and humidity as reported in other members of the *Solanum* genus (such as *S. carolinense* and *S. peruvianum*) or species under the GSI system like citrus (Webb and Williams, 1988; Stone et al., 2006; Mena-Ali and Stephenson, 2007; Enciso-Rodriguez et al., 2019; Montalt et al., 2022). Plant age is another possible confounding factor when quantifying SI, as SI breaks down in older plants of *S. tuberosum* and other *Solanum* species (Eijlander et al., 1997; Stephenson et al., 2003; Travers et al., 2004). To account for the effect of plant age, plant fertility was recorded at one month and two and a half months after the plants were transferred from tissue culture. The lines with the highest fruit weight (*S-RNase* KO, 124_001, 124_005, 124_008, and 124_137) also had the highest seed count out of all 14 lines including controls (**Figures 3B, C**) indicating larger fruits contain more seeds. Greater fruit weight observed in mature versus young plants but lack of significant difference between seed count given differences in time supports that mature plants give rise to larger fruits, but do not have increased self-fertility.

Due to the plasticity in fruit set and weight, seed count provides the most appropriate representation of SC/fertility within DRH-195. All double *HT-B* + *S-RNase* KO lines and the *S-RNase* KO were self-compatible but ranged from low to high levels of self-fertility. The majority of fruit produced in the WT and *HT-B* KO lines was parthenocarpic, except for four lines (121_008, 121_020, 121_095, and 123_001) which had low seed count with less than three seed per fruit. The 124_001 and 124_137 lines displayed enhanced seed production indicating a synergistic effect of *S-RNase* and *HT-B* in overcoming GSI. The SC reaction observed in *HT-B* + *S-RNase* KOs and the *S-RNase* KO is likely due to the gene edits in *S-RNase* preventing any functional S-RNase protein from degrading pollen RNA. However, the biochemical mechanism underlying increased fertility/enhanced seed production is unknown. Immunolocalization has shown HT-B proteins are located on vacuolar membranes containing S-RNase and higher levels of HT-B protein are found in incompatible versus compatible pollinations, supporting that HT-B plays a role in sequestration/release of S-RNase (Goldraij et al., 2006; McClure, 2006). Possible hypotheses by which HT-B enhances fertility include another unknown interaction outside GSI with HT-B (i.e. unilateral incompatibility-related mechanisms) or alternatively, by the removal of HT-B there is enhanced, but incomplete sequestration of S-RNase. Enhanced seed set compared to the *S-RNase* KO was not observed in 124_005 or 124_008 likely due to the floral mutation of petals, anther, and style fusion which inhibited pollination. These floral mutations were observed in (80%-100%) of flowers pollinated in the 121_008, 121_062 and 124_005 lines and likely contributed to 121_008 and 121_062 showing the lowest fruit set out of all the lines (p < 0.05, Tukey’s test). The *S-RNase* KO showed a higher mean seed count per fruit compared to 124_008 (p < 0.05, Tukey’s test) which may be due to modifying loci other than *S-RNase* or *HT-B* or somaclonal variation resulting from potato regeneration (**Figure 3C**) (McClure et al., 1999; O’Brien et al., 2002; Goldraij et al., 2006).

Self-incompatible plants displayed pollen tube growth from the stigma to the ovaries, contrary to the traditional GSI model (**Figure 2A**). Though the GSI model may remain representative of other species within the Solanaceae, Rosaceae, and Scrophulariaceae families, our results support the hypothesis that *S. tuberosum* DRH-195 line has a different reaction to self-pollination (Thompson and Kirch, 1992; Sassa et al., 2007; McClure et al., 2011). This incongruity is not entirely unexpected considering that prior studies in the DRH population and other angiosperm species originally hypothesized to have GSI also show complete pollen tube growth in self-incompatible lines (Seavey and Bawa, 1986; Gibbs, 2014; Peterson et al., 2016). The tomato clade has been well-studied in the Solanaceae to uncover the diversity of mechanisms contributing to SI/SC (Broz and Bedinger, 2021; Broz et al., 2021) but have not observed LSI. The potato clade has not been explored for SI/SC mechanisms despite the greater diversity. At this time complete pollen tube growth, but lack of seed production support that source of SI is derived from LSI (Seavey and Bawa, 1986; Gibbs, 2014). The LSI in DRH-195 may be due to failure to penetrate the ovules, arrested pollen tube growth within the micropyle, syngamy failure, or a post-zygotic SI mechanism (Seavey and Bawa, 1986; Gibbs, 2014).

In this study, CRISPR-Cas9 was utilized to generate KOs in *HT-B* or *HT-B* + *S-RNase* within the diploid potato line DRH195. The resulting levels of compatibility were quantitatively characterized by comparing fruit set, fruit weight, and seed count with seed count being the essential defining factor in SC. The *HT-B* + *S-RNase* double KOs 124_001 and 124_137 showed seed count levels that were over two and three times greater than the *S-RNase* KO which was the characterized as the most self-fertile *S-RNase* KO from a previous study (Enciso-Rodriguez et al., 2019). The 124_001 and 124_137 lines will provide a valuable contribution to developing self-compatible diploid potato lines in commercial cultivars and provide genetic resources to further understand SI/SC in diploid potato.

## Data availability statement

The original contributions presented in the study are included in the article/**Supplementary Material**. Further inquiries can be directed to the corresponding author.

## Author contributions

FE-R, NM-C and DD conceptualized the study. FE-R designed the CRISPR-Cas9 constructs. FE-R, SL, DZ, DD, SN, WB and CB contributed to the experimental design. SL and FE-R performed sequencing of KO lines. SL and WB performed chloroplast counting, stylar squash analysis, self-compatibility assessments, statistical design, and statistical analysis. SL, FE-R, TJ and KP performed laboratory experiments. SL, FE-R, CB, SN, NM-C, WB, and DD wrote the manuscript. The manuscript was approved of by all authors. All authors contributed to the article and approved the submitted version.
