# Supplementary material for: HT-B and S-RNase CRISPR-Cas9 double knockouts show enhanced self-fertility in diploid Solanum tuberosum
Source: Front Plant Sci. 2023 May 31;14:1151347. doi: 10.3389/fpls.2023.1151347 (PMC10264808; doi:10.3389/fpls.2023.1151347)
Supplement: Supplementary file 1 [file DataSheet_1.docx]

**Supplementary figures**


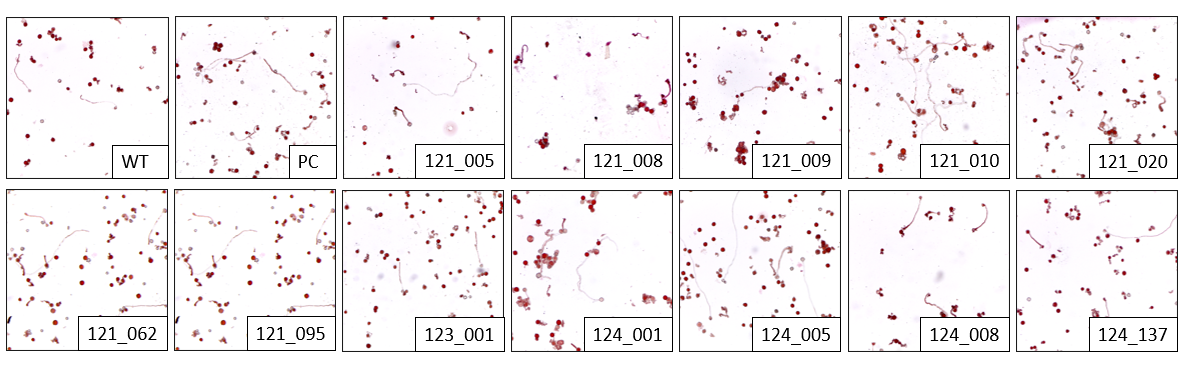


**Figure S1.** Pollen staining and germination for the T_0_ generation. All lines used in this study stained red when exposed to acetocarmine and generated pollen tube formation, indicating fertile pollen. Differences in self-compatibility are not due to infertile pollen, but rather to a stylar or other non-male determinant.


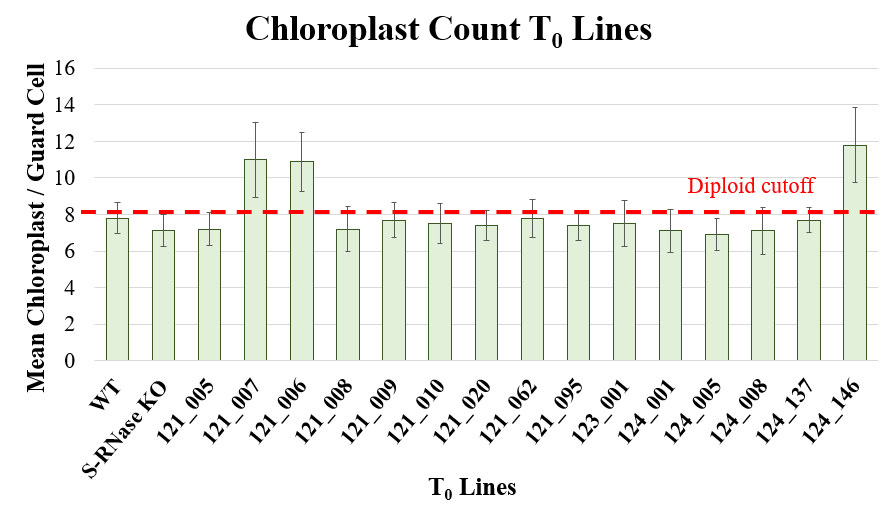


**Figure S2.** Ploidy determination via average chloroplast counts per guard cell. An average of 6-8 indicates a diploid *S. tuberosum* line. Lines selected for fertility assessment fall below the average cutoff of eight (dashed red line) chloroplasts per guard cell and are diploid. Error bars represent the standard deviation. T_0_ lines selected for study in addition to 121_007, 121_006, and 124_146 lines which all had average chloroplast per guard cell count greater than eight and were removed from the study.


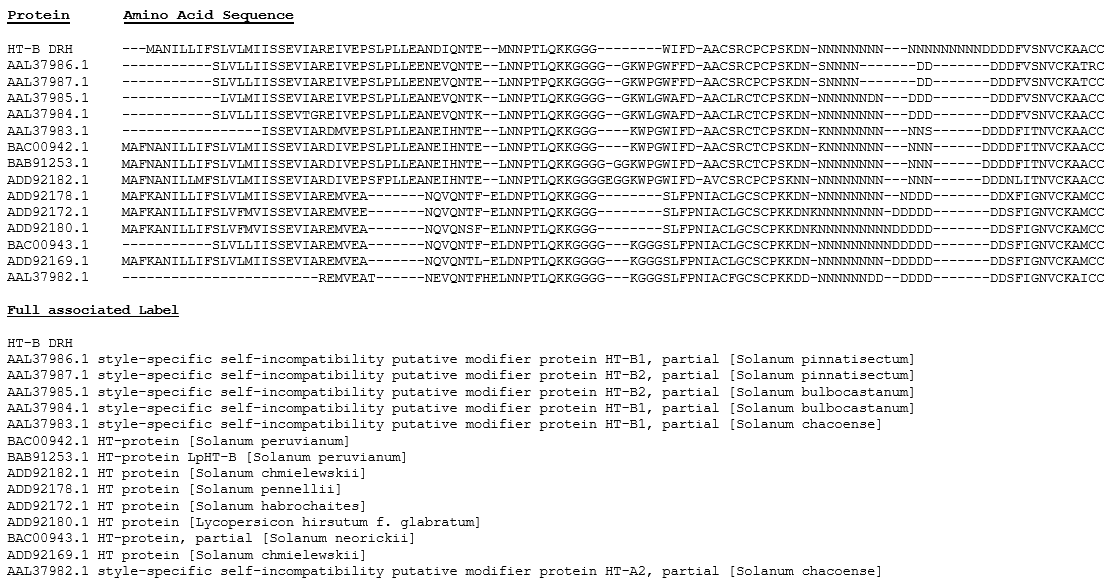


**Figure S3.** Amino acid alignment comparison between various species and accessions.

**
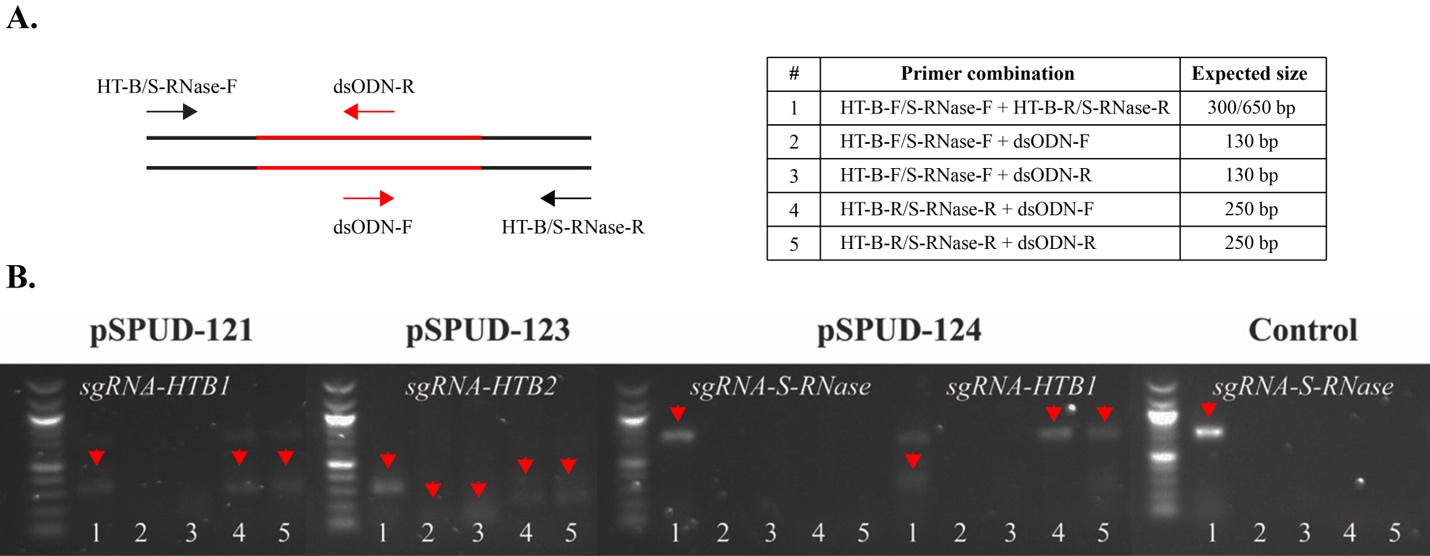
**

**Figure S4.** Detection of dsODNs integration in *HT-B* and *S-RNase* by PCR.  **A.** *HT-B*and *S-RNase* have been amplified using dsODN specific primer and gene specific primers. dsODN can be inserted in two possible directions at the target site. Five primer combinations, including a positive control (#1) were used. **B.** Four different PCR reactions were performed using single gRNAs targeting the *HT-B* gene (sgRNA-HTB1 and sgRNA-HTB2), *S-RNAse* and *HT-B* (sgRNA-S-RNAse + sgRNA-HTB1) along with a negative control (using the sgRNA-S-RNase) without nuclease but with dsODNs to account for background DSBs. A second band is seen (>700 bp for lanes 4 and 5 respectively) as a result of a dsODN insertion on each sgRNA target site, resulting in two possible bands per primer combination. A similar trend was observed for the sgRNA-HTB2 and sgRNA-S-RNase (650 bp for lane 1) combinations. The visible expected bands are indicated by red arrows.


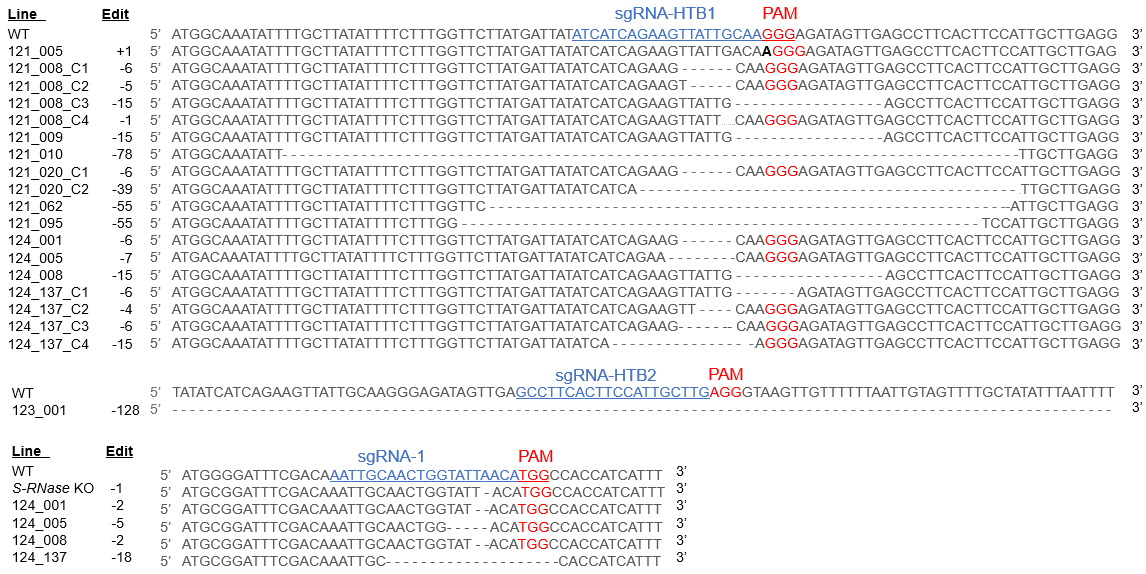


**Figure S5.** All edited sequences for 121, 123, 124, WT, and *S-RNase* KO lines.The bolded “A” in the 121_005 sequence indicates an insertion. Dashed lines indicate deleted regions.


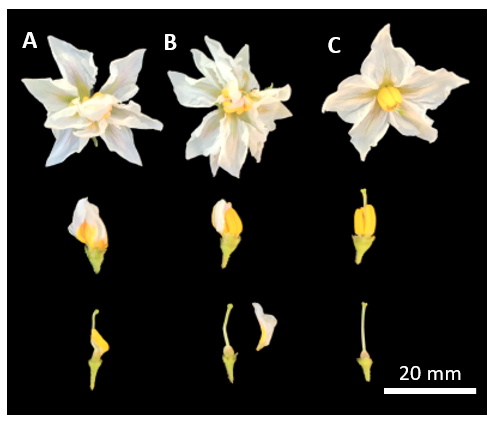


**Figure S6.** Floral mutations in some T_0_ lines. (A) Severe mutations defined as fusion of petals, anthers, and the style. Flower example from 124_005. (B) Moderate mutation defined as fusion of petals and anthers, but no fusion in the style. Flower example from 121_062. (C) No mutation, flower example from WT.
