# Supplementary material for: HT-B and S-RNase CRISPR-Cas9 double knockouts show enhanced self-fertility in diploid Solanum tuberosum
Source: Front Plant Sci. 2023 May 31;14:1151347. doi: 10.3389/fpls.2023.1151347 (PMC10264808; doi:10.3389/fpls.2023.1151347)
Supplement: Supplementary file 2 [file Table_1.docx]

**Supplementary tables**

**Table S1.** List of primers utilized in this study.

| **Label** | **Sequence** | **Description** |
| --- | --- | --- |
| HTB-F | 5' - CAACAAACTCATATAAAATGGC - 3' | *HT-B forward* primer |
| HTB-R | 5' - CTAACAACAAGCGGCTTTACA- 3' | *HT-B* reverse primer |
| S-RNase-F | 5' - ATGTTTAAATCACTGCTTACATCAAC - 3' | *S-RNase* forward primer |
| S-RNase-R | 5' - TCAGGGACGGAAAAATATTTTCCCTG - 3' | *S-RNase* reverse primer |

**Table S2.** Transformation efficiency in DRH-195 using constructs targeting the *HT-B* (pSPUD-121 and pSPUD-123) and *HT-B* + *S-RNase* (pSPUD-124) genes.

| **Construct** | **sgRNA** | | **Initial Explants** | **Transformation Events** | **Transformation**  **Efficiency (%) *** | |
| --- | --- | --- | --- | --- | --- | --- |
| pSPUD-121 | | sgRNA-HTB1 | 220 | 148 | 67 | |
| pSPUD-123 | | sgRNA-HTB2 | 600 | 330 | 55 | |
| pSPUD-124 | | sgRNA-HTB1 + sgRNA-RNase** | 600 | 250 | 42 | |
| **Percentage of recovered sprouts from selective regeneration media* | | | | | |  |
| ***Edits in either HT-B or S-RNase* | | |  |  | |  |

**Table S3.** Frequencies of chimeric alleles observed in this study.

| **Line and chimera (C) #** | **Gene Edit** | **Sequence Edit*** | **Percentage (%)**** |
| --- | --- | --- | --- |
| 121_008_C1 | -6 | TCATCAGAAG - - - - - - CAA**GGG**AGATAGTTGAG | 43% |
| 121_008_C2 | -5 | TCATCAGAAGT - - - - - CAA**GGG**AGATAGTTGAG | 25% |
| 121_008_C3 | -15 | TCATCAGAAGTTATTG - - - - - - - - - - - - - - - - - AG | 19% |
| 121_008_C4 | -1 | TCATCAGAAGTTATT-CAA**GGG**AGATAGTTGAG | 13% |
| 121_020_C1 | -6 | TCATCAGAAG - - - - - - CAA**GGG**AGATAGTTGAG | 62% |
| 121_020_C2 | -39 | TCATCA - - - - - - - - - - - - - - - - - - - - - - - - - - - - - - | 38% |
| 124_008_C1 | no edit | TCATCAGAAGTTATTGCAA**GGG**AGATAGTTGAG | 43% |
| 124_008_C2 | -15 | TCATCAGAAGTTATTG - - - - - - - - - - - - - - - - - AG | 57% |
| 124_137_C1 | -6 | TCATCAGAAGTTATTG - - - - - - - AGATAGTTGAG | 4% |
| 124_137_C2 | -4 | TCATCAGAAGTT - - - -CAA**GGG**AGATAGTTGAG | 18% |
| 124_137_C3 | -6 | TCATCAGAAG- - - - - -- CAA**GGG**AGATAGTTGAG | 9% |
| 124_137_C4 | -15 | TCA - - - - - - - - - - - - - - - -A**GGG**AGATAGTTGAG | 4% |
| 124_137_C5 | no edit | TCATCAGAAGTTATTGCAA**GGG**AGATAGTTGAG | 65% |
| **Showing regions upstream of the sgRNA and downstream of the PAM. Deletions represented by a dash.* | | | |
| ***Percentage of alleles may vary depending on cell samples taken.* | | |  |
| **PAM is in bold and red.** | |  |  |
